# Molecular characteristic of imipenem-resistant Pseudomonas aeruginosa isolated from unrinary tract infections in Southern Poland

**DOI:** 10.1186/2047-2994-4-S1-P139

**Published:** 2015-06-16

**Authors:** M Pobiega, J Maciag, A Chmielarczyk, D Romaniszyn, M Monika Pomorska-Wesolowska, G Ziolkowski, J Wojkowska-Mach, PB Heczko

**Affiliations:** 1 Jagiellonian University Medical School, Kraków, Poland; 2 Department of Dental Prophylaxis and Experimental Dentistry, Institute of Dentistry Jagiellonian University Medical College, Kraków, Poland; 3 Higher School of Medicine in Sosnowiec, Sosnowiec, Poland

## Introduction

Carbapenem-resistant *Pseudomonas aeruginosa* (PAR) has become a serious health problem worldwide. It is essential to understand its epidemiology as it may help to control the antibiotic resistance.

## Objectives

To analyze the molecular characteristics of carbapenem-resistant PAR in urinary tract infections in Southern Poland.

## Methods

Antimicrobial susceptibility testing was performed. Metallo-beta-lactamases were detected. Multidrug-resistant (MDR) was non-susceptible to one antimicrobial in ≥3 antimicrobial classes. Extensively-drug resistant strain (XDR) was susceptible to ≤2 antimicrobial classes. MLST was performed (*Curran et al *,*2004*).

## Results

The median (Q1;Q3) age was 60 years (54;69), 33.3% were females. Among 183 urine samples contained *P. aeruginosa*, 21 imipenem-non-susceptible strains were included for further analysis. MIC50 for imipenem was 12.0 mg/l. Eighteen strains (86.0%) were resistant to meropenem (MIC50=8.0 mg/l). Sixteen strains (76.0%) were resistant to doripenem. Based on the EDTA-assay, 9 (42.8%) MBL-positive isolates were identified. VIM-2 was present in three isolates. No isolates with SPM nor IMP, SIM, GIM were detected. Three (14.2%) isolates were classified as MDR, 8 as XDR (38%). MDR/XDR strains were found more often among polimycrobial infections than monomicrobial (p=0.042, OR=0.093, 95% CI 0.0085-1.00). Eight XDR strains were designated to MLST typing scheme. Four strains belonged to ST235, two strains to ST 260. The remaining two strains belonged to ST654 o ST234, respectively.

## Conclusion

This study indicated the emergence of MDR and XDR strains producing MBL. A high prevalence of imipenem-resistant strains and MBL is a critical problem and a therapeutic challenge for clinicians. Continuous surveillance is necessary to detect the presence of MBL-producing strains. No 2012/05/N/NZ7/00786.

## Disclosure of interest

None declared.

